# Antibacterial, Antihemolytic, Cytotoxic, Anticancer, and Antileishmanial Effects of *Ajuga bracteosa* Transgenic Plants

**DOI:** 10.3390/plants10091894

**Published:** 2021-09-13

**Authors:** Samina Rubnawaz, Mohammad K. Okla, Nosheen Akhtar, Imdad Ullah Khan, Muhammad Zeeshan Bhatti, Hong-Quan Duong, Mohamed A. El-Tayeb, Yahaya B. Elbadawi, Khalid S. Almaary, Ihab M. Moussa, Zahid Khurshid Abbas, Bushra Mirza

**Affiliations:** 1 Department of Biochemistry, Quaid i Azam University, Islamabad 45320, Pakistan; bushramirza@qau.edu.pk; 2 Botany and Microbiology Department, College of Science, King Saud University, Riyadh 11451, Saudi Arabia; malokla@ksu.edu.sa (M.K.O.); mali5@ksu.edu.sa (M.A.E.-T.); yalbadawi@ksu.edu.sa (Y.B.E.); Kalmaary@ksu.edu.sa (K.S.A.); imoussa1@ksu.edu.sa (I.M.M.); 3 Department of Biological Sciences, National University of Medical Sciences, Rawalpindi 46000, Pakistan; nosheenakhtar@numspak.edu.pk (N.A.); zeeshan.bhatti@numspak.edu.pk (M.Z.B.); 4 Department of Biotechnology, Abdul Wali Khan University, Mardan 23200, Pakistan; ik16092@gmail.com; 5 Laboratory Center, Hanoi University of Public Health, Hanoi 100000, Vietnam; dhq@huph.edu.vn; 6 Biology Department, College of Science, Tabuk University, Tabuk 71491, Saudi Arabia; Zahid104@yahoo.com

**Keywords:** *Ajuga bracteosa*, qPCR, FTIR, principal component analysis, antibacterial, antihemolytic, brine shrimp cytotoxicity, anticancer, cell lines, antileishmanial activity

## Abstract

Herbal and traditional medicines can play a pivotal role in combating cancer and neglected tropical diseases. *Ajuga bracteosa*, family Lamiaceae, is an important medicinal plant. The genetic transformation of *A. bracteosa* with *rol* genes of *Agrobacterium rhizogenes* further enhances its metabolic content. This study aimed at undertaking the molecular, phytochemical, and in vitro biological analysis of *A. bracteosa* extracts. We transformed the *A. bracteosa* plant with *rol* genes and raised the regenerants from the hairy roots. Transgenic integration and expression of *rolB* were confirmed by conventional polymerase chain reaction (PCR) and qPCR analysis. The methanol: chloroform crude extracts of wild-type plants and transgenic regenerants were screened for in vitro antibacterial, antihemolytic, cytotoxic, anticancer, and leishmanial activity. Among all plants, transgenic line 3 (ABRL3) showed the highest expression of the *rolB* gene. Fourier transform infra-red (FTIR) analysis confirmed the enhanced number of functional groups of active compounds in all transgenic lines. Moreover, ABRL3 exhibited the highest antibacterial activity, minimum hemolytic activity (CC_50_ = 7293.05 ± 7 μg/mL) and maximum antileishmanial activity (IC_50_ of 56.16 ± 2 μg/mL). ABRL1 demonstrated the most prominent brine shrimp cytotoxicity (LD_50_39.6 ± 4 μg/mL). ABRL3 was most effective against various human cancer cell lines with an IC_50_ of 57.1 ± 2.2 μg/mL, 46.2 ± 1.1 μg/mL, 72.4 ± 1.3 μg/mL, 73.3 ± 2.1 μg/mL, 98.7 ± 1.6 μg/mL, and 97.1 ± 2.5 μg/mL against HepG2, LM3, A549, HT29, MCF-7, and MDA-MB-231, respectively. Overall, these transgenic extracts may offer a cheaper therapeutic source than the more expensive synthetic drugs.

## 1. Introduction

In recent years, the emergence of bacterial resistance against the 3rd and 4th generation beta-lactam drugs is a big challenge. To counter this, several new drugs have been developed, and medicinal plants have also been acknowledged as a potential source of therapeutic compounds for drug design. Despite these big strides, bacterial resistance to many antimicrobials is still on the increase [1]. More recently, numerous studies have been carried out on the antibacterial activities of phenolic-rich plant extracts. However, due to the enormous wealth of plant species and phytoconstituents, the level of their investigation is still insufficient [2]. Additionally, no antimicrobial with economic potential has yet been discovered from plants. Therefore, further screening of plants could facilitate the discovery of suitable antimicrobial drugs.

Many studies ascertain the potential health benefits of plant polyphenols as naturally occurring antioxidants [3]. The human erythrocyte has been proposed as a valuable in vitro model to study the oxidant/antioxidant interaction. The erythrocyte membrane is extremely susceptible to free-radical-mediated peroxidation due to its high content of polyunsaturated fatty acids, which might be counteracted by antioxidants [4]. Thus, hemolysis is an important indicator of free-radical-induced membrane damage to the human red blood cells (RBCs) [5].

Cancer and leishmaniasis are major health problems, and the struggle to combat these disorders is a huge challenge to mankind [6]. Cancer is the second leading cause of mortality and morbidity after cardiovascular disease worldwide. The World Health Organization (WHO) has estimated that approximately 11 million people are diagnosed with cancer every year, causing 7 million deaths per year [7]. According to the latest report, 8.2 million new cases of cancer and 5.2 million deaths are reported in Asia. Most people are affected by breast cancer (13.5%), lung cancer (8%), and colorectal cancer (6.2%) [8]. Despite the huge amount spent on synthetic drug development, still many difficulties persist in cancer therapeutics. Therefore, considerable effort has centered around plants for identifying cost-effective and target-oriented chemopreventive agents [9].

Brine shrimp cytotoxicity is a simple and inexpensive bench-top bioassay used for preliminary determination of cytotoxicity of natural substances [10]. The strong correlation between the brine shrimp assay and in vitro growth inhibition of human cancer cell lines demonstrated by the National Cancer Institute (NCI, USA) is significant because it shows the value of this bioassay as a pre-screening tool for antitumor drug research. Thus, this method can be used to evaluate the toxicity of plant samples for predicting cytotoxic concentration range and cytotoxicity validation in cancer cell lines [11]. Leishmaniasis is an endemic yet neglected disease caused by multiple protozoan parasite species of the genus *Leishmania*. It is transmitted to humans by the bite of infected female sandflies in tropical and subtropical areas. This devastating parasitic infection has various clinical manifestations, including cutaneous leishmaniasis (CL), visceral leishmaniasis (VL), and mucocutaneous leishmaniasis (ML) [12]. According to WHO, 85% of CL cases are found in 10 countries, including Pakistan, while 1.7 billion people in around 98 countries are at risk of developing leishmaniasis [13]. It is speculated that leishmaniasis might contribute to carcinogenesis in immunocompromised patients and vice versa. However, the complete etiology of the underlying mechanism is not fully understood [14]. Currently available anti-parasitic drugs (pentavalent antimonials) have several disadvantages in terms of high cost, toxicity, poor prognosis, and serious side effects [15].

Natural products, particularly plants, have been used for the treatment of infectious diseases and malignancies since prehistoric times. *Ajuga bracteosa* Wall. ex Benth. is one such perennial herbal plant growing wild in hilly areas from Kashmir to Pakistan. Traditionally, root extracts of *A*. *bracteosa* are used for the treatment of diarrhea, dysentery, and inflammatory disorders, whereas the decoction of leaves and bark is used for cancer, sore throat, cough, pneumonia, and other respiratory issues [16,17]. Furthermore, this plant is also used to treat hypoglycemia, jaundice, protozoan infection, and gastric ulcer [18]. The holistic medicinal value of *A*. *bracteosa* is ascribed to the presence of phenolics, flavonoids, terpenoids, saponins, tannins, and other metabolites [19].

Unfortunately, the amount of these medicinally important metabolites is very low in naturally growing *A*. *bracteosa*. However, genetic transformation of *A. bracteosa* with *root oncogenic loci* (*rol*) genes, found in the T-DNA of *Agrobacterium rhizogenes*, offers a promising approach to enhance the production of secondary metabolites [20]. Among all *rol* genes, *rolB* is the most powerful inducer of secondary metabolism following an unknown mechanism. The *rolB* gene not only induces the large-scale production of therapeutically important products in plants but also positively regulates photosynthesis and increases plant tolerance against biotic and abiotic stresses, thus, improving plant survival [21].

Keeping this in mind, the current study was designed to analyze the expression of *rolB* genes in *A. bracteosa* plants regenerated from *rolABC* transformed hairy roots. Furthermore, the effect of *rol* genes on the production of secondary metabolites and various biological activities of *A. bracteosa* plants was also investigated.

A variety of techniques can be used to detect phytochemical entities of medicinal plants, e.g., spectroscopy. Fourier-transform infrared (FTIR) spectroscopy is a simple, cheap, and rapid tool to characterize functional groups and chemical bonds in biological samples [22]. FTIR coupled with principal component analysis (PCA), a multivariant technique, describes a correlation between different species and multiple samples of the same species based on their spectral profiles [23].

In the present study, genetic integration of the *rolB* gene in *A*. *bracteosa* was validated by conventional polymerase chain reaction (PCR), and its real-time expression was checked by quantitative real-time PCR (qPCR). FTIR coupled PCA was applied to identify medicinally important functional groups in *A*. *bracteosa*. This study also assessed the antibacterial, anti-hemolytic, cytotoxic activity against brine shrimps and antileishmanial activity of crude extracts. The dose-dependent anticancer effect of *A*. *bracteosa* against human liver cancer cell lines (HepG2, LM3), human colon cancer cell line (HT-29), human lung cancer cell line (A549), and human breast cancer cell lines (MCF-7, MDA-MB-231) were also evaluated.

## 2. Results

In this study, three independent transgenic lines of intact *Ajuga bracteosa* plants (ABRL1-3) were successfully regenerated from transgenic hairy roots. These regenerated plants presented distinct morphological variability from untransformed tissue cultured wild-type (WT) *A*. *bracteosa* plants. WT plants were tall and soft in texture with prominent nodal regions, whereas all transgenic regenerants were abnormally dwarf with short internodes and bushy appearance. Leaves were wrinkled and visibly lacking midrib.

### 2.1. Molecular Analyses of Ajuga bracteosa Plants

**Figure 1:**
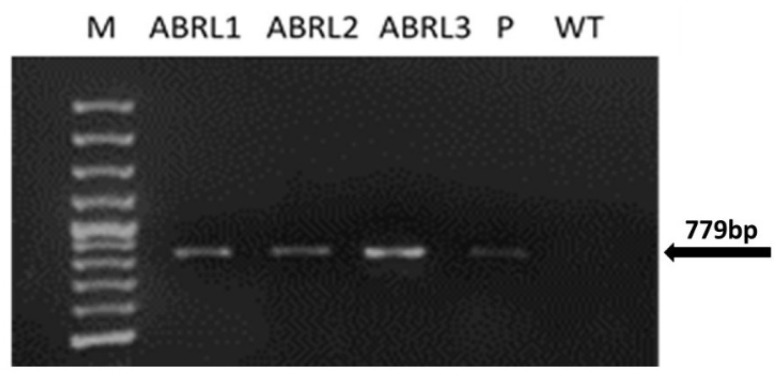
PCR amplification with *rolB* specific primers. *Lane M* represents 100 bp DNA marker (Fermentas); *ABRL1-3* are transgenic lines of *A*. *bracteosa*; *WT* stands for wild-type tissue cultured plants; *P* corresponds to plasmid DNA.

PCR performed with *rolB* primers showed the presence of a 779 bp amplicon in regenerated plantlets, as shown in Figure 1. Similar amplified products were obtained from LBA-9402 plasmid DNA. No such band was found in WT plants.

**Figure 2:**
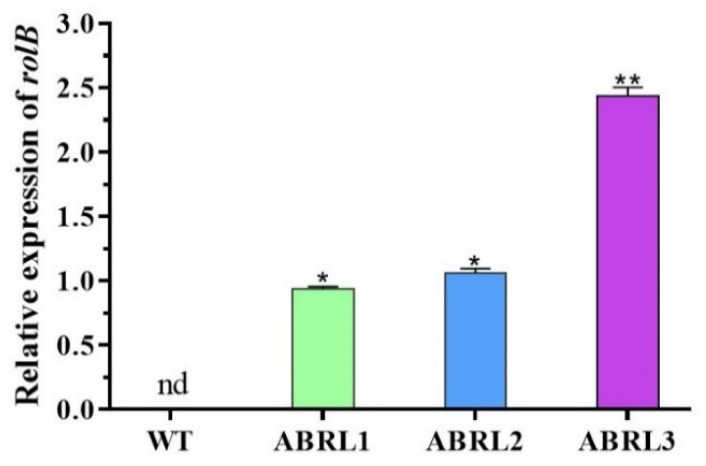
Relative expression of *rolB* gene in wild type and three independent transgenic lines of *A*. *bracteosa*. The expression level was normalized with the *β-actin* and *18s* reference genes. Data are expressed as mean ± S.D. (** *p* *<* 0.01, * *p* *<* 0.05), nd = not detected.

The expression of the *rolB* gene in wild type and transgenic regenerants of *A*. *bracteosa* was assessed by qPCR. We found no expression of the *rolB* gene in the WT plants. Overall, transgenic line 3 (ABRL3) showed the most significant expression (** *p* *<* 0.01) of the *rolB* gene, followed by transgenic line 2 (ABRL2) and transgenic line 1 (ABRL1), as given in Figure 2.

### 2.2. FTIR Coupled with PCA

**Figure 3:**
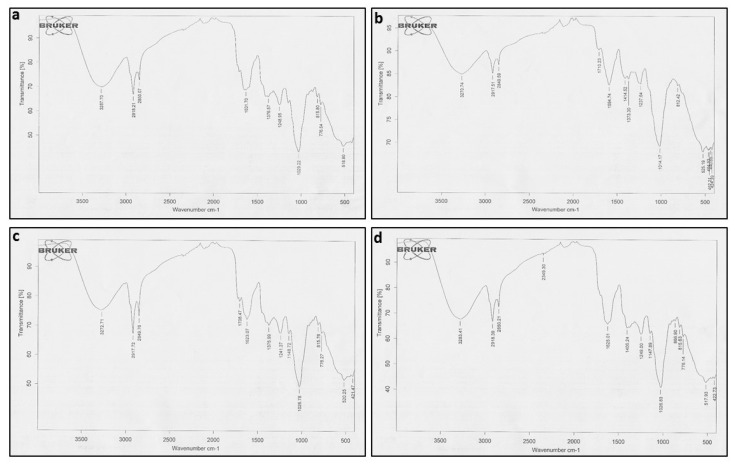
FTIR spectra of different lines of *Ajuga bracteosa* (**a**) WT (**b**) ABRL1 (**c**) ABRL2 (**d**) ABRL3.

FTIR analysis was used to determine the functional groups of active compounds present in both transformed and wild-type plant extracts of *A*. *bracteosa*. The wavelength of light absorbed is a salient feature of the chemical bonds, as can be seen in the annotated spectra (Figure 3a–d).

These spectra revealed that ABRL1 has 11 major peaks between 525.19 and 3270.74 nm, ABRL2 has 12 (520.25–3272.71 nm), ABRL3 has 13 (517.93–3283.41 nm), while WT has 10 peaks (518.90–3287.70 nm). All the crude extracts represented somewhat similar functional groups except Thiol/mercaptan (S-H) at 2349.30 nm, only found in ABRL3 and Ether group found in ABRL2 and ABRL3. Peak values of absorption spectra, along with their expected functional groups, are given in Table 1.

**Figure 4:**
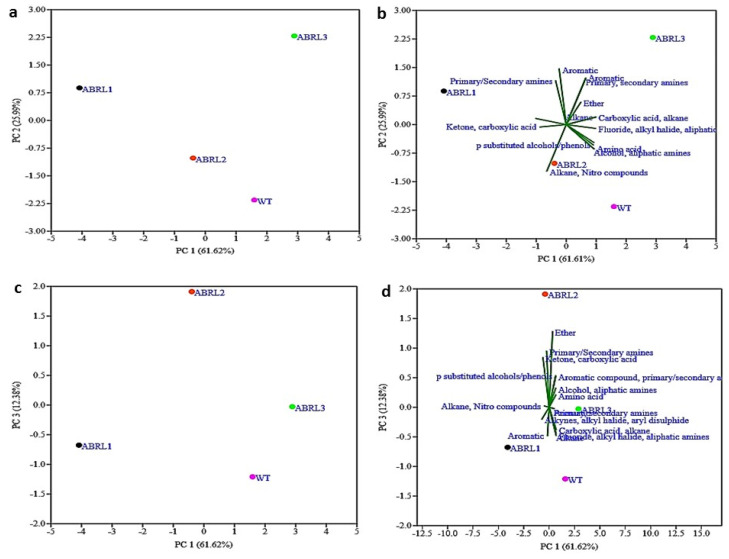
Principal component analysis (PCA) of functional groups detected through FTIR (**a**) scatter plot of samples in PC 1 vs. PC 2 (**b**) scatter plot of functional groups in PC 1 vs. PC 2 (Biplot) (**c**) scatter plot of PC 1. vs. PC 3 (**d**) scatter plot of functional groups in PC 1 vs. PC 3 (Biplot).

The multivariate statistical analysis technique, PCA, successfully explains the variability in each data set. In the current study, PCA was used to evaluate the extent of similarities or differences between multiple plant samples based on the number of detected functional groups. Here, PCA recognized three significant components: principal component 1 (PC1) explained 61.62% of the variance, while principal component 2 (PC2) had a variation of 25.99%. Principal component 3 (PC3) showed 12.38% of the variability. The combination of PC1 and PC2, as well as PC1 and PC3, categorized the four used plant samples into distinctive regions (Figure 4a,c). This confirmed that significant diversity of functional groups did exist in all samples. A biplot marked a clear relationship among different lines of *A*. *bracteosa* and their functional groups (Figure 4b,d).

### 2.3. Antibacterial Activity

All extracts of *A. bracteosa* were screened for their antimicrobial activity against four bacterial strains, namely, *Micrococcus luteus*, *Staphylococcus aureus, Enterobacter aerogenes*, and *Escherichia coli* at a concentration of 100 µg/mL. The results of antibacterial activity are shown in Table 2. Among all the strains tested, the plant extracts were more active against *S. aureus* and *E. coli* with ABRL3 yielding the highest zone of inhibitions (ZOIs) (16.2 ± 0.7 and 16.2 ± 0.5 mm, respectively) at 100 µg/disc. Overall, ABRL3 showed maximum antibacterial activity (8.2 ± 0.3–16.9 ± 0.7 mm ZOI), and its ZOIs were comparable to the standard kanamycin (23.7 ± 1–26.2 ± 1 mm). DMSO, negative control, showed no inhibition against any bacterial strain.

### 2.4. The Antihemolytic Activity of Plant Extracts

The antihemolytic activity of crude extracts of *A*. *bracteosa* was screened against normal human erythrocytes. WT and three transgenic lines exhibited a differential pattern of hemolytic effect towards human erythrocytes. The result indicated that the ABRL3 exhibited minimum hemolytic activity (CC_50_ = 7293.05 ± 7 µg/mL), whereas WT extracts showed the highest hemolytic activity (CC_50_ = 1952.63 ± 12 µg/mL). Lysis of erythrocytes was found to be increased with an increase of extract concentration (Table 3).

### 2.5. Brine Shrimp Lethality Activity

Brine shrimp assay was performed to monitor the cytotoxic potential of active compounds in different concentrations of extracts. Cytotoxicity results of all extracts of *A. bracteosa* are summarized in Table 4. The lower the LD_50_ value, the more toxic the substance. In vitro grown WT plant showed the highest LD_50_ of 75.6 ± 9 μg/mL, whereas ABRL1 and ABRL2 showed the lowest LD_50_ values of 39.6 ± 4 μg/mL and 41.62 ± 2 μg/mL, respectively, indicating a high level of toxicity in these extracts.

### 2.6. Plant Extracts Inhibited Cancer Cell Growth in a Concentration-Dependent Manner

**Figure 5:**
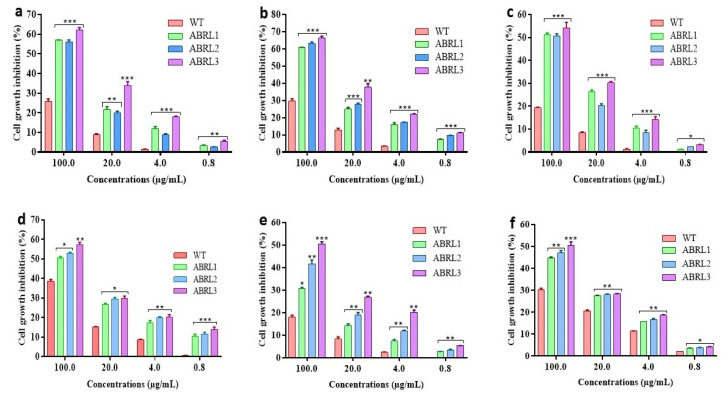
Cytotoxic activity of *Ajuga bracteosa* against (**a**) HepG2, (**b**) LM3, (**c**) HT-29, (**d**) A549, (**e**) MCF-7, and (**f**) MDA-MB-231. WT = wild type untransformed *A. bracteosa* plants; ABRL1–3 = transgenic lines 1–3 of *A. bracteosa*. Each value represents mean ± SD (*n* = 3). * *p* < 0.05, ** *p* < 0.01, and *** *p* < 0.001 are statistically significant data.

To test the potent anticancer activity of the crude extracts of the *A. bracteosa*, HepG2, LM3, HT-29, A549, MCF-7, and MDA-MB-231 cancer cells were treated with different concentrations of plant extracts. In the present cytotoxicity analysis, all the plant extracts inhibited the cell growth with different efficacy depending on the specific plant extract and the cancer cell line tested. As can be seen in Figure 5a–f, the inhibition of cell growth was also in a concentration-dependent manner. Nevertheless, all the transgenic line extracts were most efficient against human liver carcinoma cells (HepG2, LM3), human lung adenocarcinoma cells (A549), and human colon carcinoma cells (HT-29) growth, even at lower concentrations.

The IC_50_ values of WT and transgenic lines of *A*. *bracteosa* against selected cell lines are represented in Table 5. Among all the tested samples, ABRL3 showed the lowest IC_50_ of 57.1 ± 2.2 µg/mL, 46.2 ± 1.1 µg/mL, 72.4 ± 1.3 µg/mL, 73.3 ± 2.1 µg/mL, 98.7 ± 1.6 µg/mL, and 97.1 ± 2.5 µg/mL against HepG2, LM3, A549, HT29, MCF-7, and MDA-MB-231, respectively.

### 2.7. Antileishmanial Activity

All the crude extracts were tested at three concentrations (1000, 500, and 250 µg/mL) for the cytotoxic activity against leishmanial promastigotes (Table 6). ABRL3 showed the most promising antileishmanial activity with an IC_50_ of 56.16 ± 2 µg/mL, followed by ABRL2 (IC_50_ = 77.53 ± 7 µg/mL), ABRL1 (IC_50_ = 163.04 ± 8 µg/mL), and WT (IC_50_ = 313.99 ± 6 µg/mL). Amphotericin B was used as a positive drug that displayed excellent cytotoxicity with an IC_50_ of 0.01 µg/mL. DMSO (negative control) did not present any activity whatsoever.

## 3. Discussion

Some bacterial species have a unique capability to mediate inter-kingdom DNA transfer by integrating and expressing their genes into the plant genome. *Agrobacterium rhizogenes*, one such example, has been readily utilized in plant genetic manipulation techniques to improve the production of pharmaceutically important secondary metabolites [24].

*Agrobacterium*-mediated transformation and generation of hairy roots have been reported for various species of the *Ajuga* genus [20,25,26,27]. Hairy roots are considered stable and easy-to-cultivate variants capable of producing special secondary metabolites by transformation with wild-type *A. rhizogenes*.

They contain a wide range of metabolites either comparable or sometimes even in higher amounts than the mother plant. However, certain metabolites show organ specificity for their synthesis and optimal activity, thus, requiring organogenesis from hairy roots [28].

In the current study, multiple transformation events generated 10 independent transgenic hairy root lines of *Ajuga bracteosa*. Based on their growth rate and metabolic content, only three such lines were selected to regenerate intact plants via somatic embryogenesis. These putatively transgenic plants exhibited distinct morphological alterations characteristic of *rolABC* transformed plants [20,29]. All the investigated transgenic lines displayed stunted growth and compact posture with reduced apical dominance. This supports the well-established paradigm of the strong association of *rolB* and *rolC* to stunted growth, and this has also been reported in tomato [30], potato, tobacco [31], and many species of *Artemisia* L. [29,32,33].

PCR analysis of the *rolB* gene in transgenic regenerants of *A*. *bracteosa* revealed a successful integration of T-DNA in the plant genome. While real-time qPCR confirmed the relatively higher expression of the *rolB* gene in all transgenic lines. The *rolABC* oncogenes not only regulate hormone-controlled morphogenesis but also powerfully induce plant secondary metabolism [34]. Previously, we found enhanced expression of metabolic pathway genes under the influence of *rolABC* in *A*. *bracteosa* regenerated plants [35]. This enhanced expression can be linked with significantly higher amounts of several polyphenols, as shown in our previous study [36]. Here, among all the tested plants, ABRL3 displayed the highest phenolic content, flavonoid content, antioxidant capacity, radical scavenging, and metal chelating power. Previously, *rolB* genes improved the antioxidants lycopene and ascorbic acid in transgenic tomato plants [30]. Moreover, *rolB* and *rolC* transgenic-*Artemisia carvifolia* plants showed an increased amount of Artemisinin, a natural sesquiterpene lactone utilized against malaria and as an anti-cancer agent [32]. Likewise, *rolC* is reported to increase antioxidant, anti-inflammatory, antidepressant, and analgesic properties in lettuce [37]. Further, *rolA* and *rolC*-transgenic tissues exhibit increased production of anthraquinones [38,39], ginsenosides [40], resveratrol [41], and nicotine [42]. These effects are probably due to the influence of *rol* genes on the increased transcription of *isochorismate synthase* gene, calcium-dependent protein kinase gene expression, activation of genes encoding MYB and bHLH transcription factors, and many other genes involved in biosynthetic pathways [21].

In the current study, FTIR analysis revealed the presence of amines, alkanes, alcohols, aromatic compounds, ketones, phenolics, amino acids, and carboxylic acids in crude extracts. The absorption peaks between 1700–3300 cm^−1^, corresponding to the O–H group, indicate the presence of medicinally important alcohols, acids, phenols, and their derivatives [43]. The IR spectra at 1147 cm^−1^ and 1148 cm^−1^, representing ether R=C–O–C, were only found in ABRL2 and ABRL3. Moreover, C=O stretching at 1708 cm^−1^ in ABRL2 and ABRL2 is due to the derivatives of gallic acid and other tannins. The absorption at 1200–1400 cm^−1^ shows the presence of a flavonoids-C ring [44]. FTIR spectra coupled with PCA revealed the similarities and differences between multiple lines of *A*. *bracteosa* based on the presence of functional groups and absorption spectra.

Antimicrobial resistance poses a serious threat to the effective treatment of an ever-increasing range of infections. This increasing resistance has created a need to develop new antimicrobial agents. In the last years, a rational approach to deal with antibiotic resistance problems using a combination therapy combining conventional antibiotics, plant extracts, and essential oils has been proposed [45,46,47]. In this study, we explored the potential antibacterial activity of *A*. *bracteosa* crude extracts. For this, we used four bacterial strains *M. luteus*, *S. aureus, E. aerogenes*, and *E. coli.* Our investigation exhibited moderate antibacterial activity of WT as well as transgenic line extracts. Each extract acted differently and showed variation in the antibacterial activity against each bacterial strain. Overall, the following tendency of microbial sensitivity to plant extracts was observed: *S. aureus > E. coli > M. luteus > E. aerogenes*. Previously, 14, 15-dihydroajugapitin and 8-*o*-acetylharpagide isolated from the methanolic extracts of aerial parts of *A*. *bracteosa* showed significant antibacterial activity [48]. The synergistic effect of phytochemicals of *A*. *bracteosa* against different bacterial pathogens is also evident from published literature [49,50,51]. Various data show that antibacterial mechanisms of polyphenols primarily involve inhibition of synthesis of bacterial DNA and RNA, inhibition of cytoplasmic membrane function, and interaction with some crucial bacterial enzymes [2].

The antihemolytic assay aimed to assess whether *A. bracteosa* prevented 2,2′-azobis (2-amidinopropane) dihydrochloride (AAPH)-induced oxidative damages to the erythrocyte membrane or not. Here, we observed that the incubation of RBCs with the *A*. *bracteosa* crude extracts did not induce hemolysis, indicating that these extracts are non-toxic and harmless for the cells. We also found that all transformed plant extracts significantly protected the erythrocyte membrane from hemolysis in a concentration-dependent manner. It is speculated that antioxidants, particularly polyphenols, present in the extract are mobilized to fight off the oxidant attack by binding to the RBC membrane matrix near tryptophan residues. They protect the integrity of RBCs, resulting in the delay of hemolysis [52,53]. Moreover, Rehman et al. (2015) [50] successfully demonstrated the dose-dependent anti-lipid peroxidation activity of *A*. *bracteosa* extracts, which also accounts for the antihemolytic potential of this plant. However, the antihemolytic activity of *A. bracteosa* has never been reported earlier.

Brine shrimp assay was performed to check the cytotoxic potential of plant extracts. Based on the results, all the extracts of *A*. *bracteosa* showed significant cytotoxicity (LD_50_ < 100 μg/mL) against brine *shrimp nauplii* eggs. Among all the tested extracts, ABRL1 showed the lowest LD_50_ value (39.6 ± 5 μg/mL), followed by ABRL2 (41.62 ± 2 μg/mL), ABRL3 (43.62 ± 5 μg/mL), and WT (75.6 ± 9 μg/mL). Previously, several other researchers also reported promising brine shrimp cytotoxic activity of crude extracts of *A*. *bracteosa* in different solvent systems. Rehman et al. [50] reported that the *n*-hexane fractions of *A. bracteosa* displayed an LD_50_ of 370.6 μg/mL, whereas Imran et al. (2021) [54] described significantly higher cytotoxic activity in ethyl acetate extracts of *A. bracteosa*. Moreover, Zehra et al. (2017) [49] noted the most potent brine shrimp cytotoxicity in ethanolic, acetone-distilled water, methanol-distilled water extracts of *A. bracteosa* with the LD_50_ of 46.72 ± 0.18, 51.63 ± 1.18, and 63.52 ± 1.70 μg/mL, respectively. These results confirm the presence of cytotoxic substances in the crude extracts of *A. bracteosa*.

At present, the undesirable clinical consequences of synthetic anti-cancer drugs and other chemopreventive measures have forced researchers to turn to natural products. In recent years, several studies have demonstrated the anti-cancer potential of plant extracts. Their beneficial effects are due to a complex interplay of the composite mixture of compounds present in the whole plant rather than constituent single agents alone [55]. Keeping this, we investigated the anticancer properties of *A*. *bracteosa* against various cancer cell lines. Cell growth inhibition of all six cancer lines, including HepG2, LM3, HT-29, A549, MCF-7, and MDA-MB-231, was measured after treatment with crude extracts of wild-type plants and transgenic regenerants. All plant extracts were found to be more effective against HepG2, LM3, HT-29, and A549 than MCF-7 and MDA-MB. Previously, the methanolic extracts of areal parts of *A*. *bracteosa* demonstrated significant cytotoxicity against MCF-7 with an IC_50_ of 10 μg/mL [56]. In another study, the polar extracts of *A*. *bracteosa* exhibited significant activity against the human leukemia cell line (TPH-1), whereas nonpolar and moderately polar extracts were more effective against the MCF-7 cell line [49]. This is in accordance with our results as all our extracts (except ABRL3) were least effective against MCF-7 and MD-MB-231, showing an IC_50_ of more than 100 μg/mL due to their higher polarity. Thus, we found a dose-dependent anticancer effect of *A*. *bracteosa* against human liver cancer, colon cancer, lung cancer, and human breast cancer cell lines. The traditional use of *A*. *bracteosa* against sore throat, cough, diarrhea, dysentery, and digestive disorders [57] supports these findings. Furthermore, Yousaf et al. (2018) [19] reported the antiviral activity of methanolic extracts of *A*. *bracteosa* in a time-dependent manner. These extracts decreased the hepatitis-C viral count by 75% following 48 h treatment of HCV-infected HepG2 cells.

Herbal plants are the best alternatives in the development of new antileishmanial agents due to their selective action against parasites without reducing the host cell viability [15]. This research aimed to explore the therapeutic effects of *A*. *bracteosa* extract as an anti-parasitic herbal drug. We found the significant cytotoxic activity of *A*. *bracteosa* extracts formulations against leishmanial promastigotes. The achieved results demonstrated that ABRL3 was the most promising antileishmanial agent followed by ABRL2, ABRL1, and WT extracts. Our findings are in agreement with some of the previous studies reporting the antileishmanial activity of *A*. *bracteosa* crude extracts. Dose-dependent cytotoxicity was observed with a significant mortality rate of 63.18 ± 2.29 and 58.44 ± 1.61 for n-hexane, 57.20 ± 1.19 and 48.39 ± 1.09 for ethanolic extracts of *A. bracteosa* at 1000 μg/mL for promastigote and amastigotes form of the leishmanial parasite, respectively [54]. Similarly, n-hexane, n-hexane-ethyl acetate, chloroform, and *n*-hexane-ethanol extracts of *A. bracteosa* showed excellent leishmanicidal activity with the IC_50_ of 4.69 ± 0.01, 12.16 ± 0.02, 28.62 ± 0.03, and 40.1 ± 0.02 μg/mL, respectively [49]. This information can be used to isolate and characterize the pure compounds from *A. bracteosa* as a source of antileishmanial drugs.

## 4. Material and methods

### 4.1. Plant Source

Fresh, green, viable plants were collected from Islamabad, Pakistan. The sample was formally identified, and a voucher specimen (numbered HPM-460) was placed in the departmental herbarium. The collected plants were surface sterilized, and tissue cultured on Murashige and Skoog (MS) medium. Transgenic hairy roots were generated through *rolABC* genes of *Agrobacterium rhizogenes* (LBA-9402) mediated transformation. Intact plants were regenerated from transgenic hairy roots by following the previously optimized method in our lab [20].

### 4.2. Confirmation of Genetic Integration by PCR and RT-PCR

Genomic DNA was isolated from three independent transgenic lines and wild plants of *A*. *bracteosa*. For genomic DNA isolation, the cetyl trimethyl ammonium bromide (CTAB) method [58] was employed. The Alkali lysis method [59] was used to isolate the plasmid DNA of *A. rhizogenes*. Conventional PCR was used to confirm the presence of the *rolB* gene in putatively transformed and regenerated plants. The recipe for PCR reaction mixture preparation and amplification conditions for the detection of the *rolB* gene were already optimized and used accordingly [60]. The primer sequences used for the *rolB* gene are presented in Table 7.

The TRIzol^®^/ice-based method [61] was employed to isolate RNA from wild-type plants and transgenic lines for expression analysis of the *rolB* gene. Then, the Viva cDNA synthesis kit (Vivantis cDSK01-050) was used to synthesize cDNA from 1 μg of isolated RNA by reverse transcription. This whole procedure was performed by following the manufacturer’s instructions, and synthesized cDNA was stored at −20 °C for further analysis.

Before performing the real-time experiment, the qPCR conditions were optimized for genes encoding *rolB*, *β*-*actin,* and *18s*, accordingly. The gene-specific primers used for the amplification reaction are given in Table 1. The qPCR was conducted using a Mic PCR machine (BioMolecular Systems, Upper Coomera, QLD, Australia) with a 1X Eva Green PCR master mix (Phenix Research Products, Candler, NC, USA). For real-time qPCR, a 1:10 dilution of cDNA was used. The reaction conditions for qPCR were as follows: an initial cycle of denaturation for 12 min at 95 °C, followed by 40 cycles each of denaturation for 15 s at 95 °C, primer annealing for 20 s at 60 °C (for all genes), and elongation for 20 s at 72 °C. Three biological samples were analyzed, while three technical replicates were used for each biological sample. The melting curve of amplicons confirmed the absence of primer dimers at the end of each run. The relative gene expression levels were normalized with the endogenous reference genes (*β*-*actin* and *18s*) [62].

### 4.3. Crude Extract Preparation

Whole plants of WT and three independent transgenic lines (ABRL1, 2, and 3) of *A*. *bracteosa* were taken. Any growth media attached to the plants was removed by rinsing with water. Washed plants were shade dried for a week. Dried plants were ground into powder form, and 1 g powdered material was blended with a mixture of methanol: chloroform (1:1; 5 mL). This was allowed to set for 1 h, then the mixture was sonicated for 10 min, followed by 20 min of shaking, and the process was repeated three times. Finally, the plant extracts were filtered, and pooled filtrates were dried, weighed, and stored at room temperature for downstream analysis.

### 4.4. Identification of Functional Groups by Fourier Transform Infra-Red (FTIR) Spectroscopy

FTIR spectroscopy analysis of *A*. *bracteosa* crude extracts was performed to determine structural modifications (functional groups) between wild-type and transgenic plants through the identification of chemical bonds of phytoactive compounds. FTIR analysis was conducted using Perkin-Elmer Tensor 27 FTIR spectrophotometer (Bruker, Waltham, MA, USA) in the range of 4000–400 cm^−1^ with a resolution of 1 cm^−1^ and intensity mode as % Transmittance. Spectra were taken in triplicate for each sample without previous treatment [44]. Principal component analysis (PCA) was performed by using PAST 4.03 statistical software.

### 4.5. Antibacterial Activity

The antibacterial activity of the crude extracts of *A*. *bracteosa* was evaluated by the disc diffusion method [49]. In this experiment, two Gram-positive bacterial strains, *Micrococcus luteus* (ATCC 10,240) and *Staphylococcus aureus* (ATCC 6538), and two Gram-negative strains, *Enterobacter aerogenes* (ATCC 13,048) and *Escherichia coli* (ATCC 15,224), were pre-cultured in nutrient broth for 24 h at 37 °C. Then, each refreshed bacterial strain was inoculated to the nutrient agar medium at 45  °C, poured into sterile petri plates, and allowed to solidify. After that, 5 μL of the crude extracts with a final concentration of 100 μg/mL was poured on sterile filter paper discs (4 mm) and placed on nutrient agar plates. On each plate, kanamycin (4 mg/mL. Sigma-Aldrich, St. Louis, MO, USA) was used as positive control and DMSO (Dimethylsulfoxide, Sigma-Aldrich, St. Louis, MO, USA) as a negative control. The assay was performed in triplicate and the plates were incubated at 37  °C for a period of 24 h. The antibacterial activity of the extracts was determined by measuring the diameter of zones showing complete inhibition (mm) with the help of the Vernier caliper. An inhibition zone ≥ 10 mm in diameter was considered active for the tested samples and further screening was done by three-fold micro broth dilution method to determine the minimum inhibitory concentration (MIC). Briefly, the stock solution (40 mg/mL) of each active sample (inhibition zone ≥ 10 mm) was used to prepare the master plate of concentration 8 mg/mL in sterile Mueller Hinton broth (MHB). Then these samples were serially diluted in a 96-well microtiter plate with sterile MHB to obtain the final concentration ranging from 7.41 to 200 μg/mL. Subsequently, a standardized inoculum (5 × 10^4^ CFU/mL) for each bacterial strain was poured into each well. These plates were then kept at 37 °C for overnight incubation. The lowest concentration at which the extract exhibited visible growth inhibition was designated as its MIC.

### 4.6. Anti-Hemolytic Assay

To evaluate the possible hemolytic effect of crude extract of transformed and wild-grown *A. bracteosa*, an anti-hemolysis assay was performed. For this, hemolysis was induced by AAPH radicals [63]. Briefly, 5 mL blood was obtained from a unanimous human donor by venipuncture in an Ethylenediaminetetraacetic acid (EDTA) tube. The collected blood was diluted with phosphate buffer saline (PBS; 1:3). The erythrocytes from the blood were collected through centrifugation at 1500× *g* for 10 min. The pellet was washed twice with PBS (Sigma-Aldrich, St. Louis, MO, USA) while the supernatant was discarded. Washed erythrocytes were further diluted with PBS and treated with plant extracts at three different concentrations (1000, 500, and 250 µg/mL). AAPH treated blood was used as a positive reference while PBS was taken as the negative control. The mixtures were allowed for an incubation period of 1 h at 37 °C. Final centrifugation was given to the samples at the above-mentioned conditions. For hemolysis measurement, 200 µL of the supernatant from each treated sample was taken and absorbance was recorded at 570 nm. The percent hemolysis for each extract was calculated using the following formula.

(1) $$\mathrm{Percent}\;\mathrm{hemolysis}=\frac{A570\mathrm{nm}\;\mathrm{of}\;\mathrm{Sample}-A570\mathrm{nm}\;\mathrm{of}\;\mathrm{Negative}\;\mathrm{control}}{A570\mathrm{nm}\;\mathrm{of}\;\mathrm{Positive}\;\mathrm{control}-A570\mathrm{nm}\;\mathrm{of}\;\mathrm{Negative}\;\mathrm{control}}\times 100$$

CC_50_ (50% cytotoxic concentration) values were calculated from percent hemolysis.

The study protocol complied with the Helsinki Declaration. Study approval (BEC-FBS-QAU2019-157) was obtained from the Ethical Review Committee, Quaid-i-Azam University. Islamabad. Informed consent was obtained from persons who participated in the study.

### 4.7. Brine Shrimp Cytotoxicity Assay

A brine shrimp assay was performed to check the cytotoxicity of plant extracts [64]. A stock solution (40 mg/mL) of each test sample was prepared. From this stock solution, further dilutions (200, 66, and 21.8 µg/mL) were made. Then, 25 µL from each dilution was taken in a transparent glass vial and mixed in 2 mL of seawater. Ten live shrimps of *Artemia salina* were aspirated by gentle pipetting and transferred to each vial, raising the volume to 5 mL by adding seawater. This reaction mixture was kept under illumination at room temperature for 24 h. Then, the number of shrimps alive was counted with the help of a 3× magnifying glass. The experiment was performed in triplicate. Doxorubicin (4 mg/mL, Sigma-Aldrich, St. Louis, MO, USA) was used as positive control, while DMSO was the negative control.

(2) $$\mathrm{Percent}\;\mathrm{mortality}=\frac{\mathrm{No}.\;\mathrm{of}\;\mathrm{shrimps}\;\mathrm{alive}\;\mathrm{in}\;\mathrm{test}-\mathrm{No}.\;\mathrm{of}\;\mathrm{shrimps}\;\mathrm{alive}\;\mathrm{in}\;\mathrm{control}}{\mathrm{No}.\;\mathrm{of}\;\mathrm{shrimps}\;\mathrm{alive}\;\mathrm{in}\;\mathrm{control}}\times 100$$

The median lethal dose (LD_50_) of the test samples with ≥50% mortality was calculated using table curve 2D v5.01 software.

### 4.8. Antiproliferative Activity

#### 4.8.1. 3-(4,5-Dimethylthiazol-2-yl)-2,5-Diphenyl-2H-Tetrazolium Bromide (MTT) Assay

An MTT assay was performed to assess the antiproliferative activity of plant extracts under study. MTT assay is a colorimetric technique based on the ability of the living cells to transform tetrazolium salt 3-(4,5-dimethylthiazol-2-yl)-2,5-diphenyltetrazolium bromide (MTT) by the mitochondria into a formazan, a characteristic purple precipitate [65]. For that purpose, six cancer cell lines were used, including HepG2 and LM3 (derived from hepatic carcinoma), HT-29 (derived from colon cancer cells), A549 (derived from lung adenocarcinoma), and MCF-7 and MDA-MB-231 (derived from breast carcinoma). All these cell lines were purchased from ATCC (Manassas, VA, USA).

#### 4.8.2. Sample Preparation for MTT Assay

Dried crude extracts of different plant samples were dissolved in 5% (*v*/*v*) DMSO (20 mg/mL). Ellipticine (Sigma-Aldrich, St. Louis, MO, USA) was used as reference positive control, while the cells treated only with the solvent (5% DMSO) were used as a negative control.

#### 4.8.3. Maintenance of Cell Cultures

All the cell lines were cultured in RPMI 1640 medium supplemented with 10% heat-inactivated fetal bovine serum (Bio West, Miami, FL, USA) and 1% antibiotic-penicillin/streptomycin (Invitrogen, Carlsbad, CA, USA). Cells grown as monolayer cultures in T-75 flasks Costar were subcultured twice a week at 37 °C and 5% CO_2_ in a humidified atmosphere and maintained at low passage number (5 to 20).

For the cytotoxicity assay, 4–6 × 10^4^ cells/well for different cell lines were seeded in a 12-well plate. Cells were grown for 24 h, and then plant extracts were added at different concentrations (100, 20, 4, and 0.8 µg/mL) to the medium in the respective well, analyzing the toxicity after 72 h. All the conditions were run in triplicate.

#### 4.8.4. Assay Procedure

The growth medium was removed and 0.63 mM of MTT and 18.4 mM of sodium succinate (Sigma-Aldrich, St. Louis, MO, USA) were added to 1 mL of fresh culture medium, and the cells were incubated for 3 h at 37 °C. Then, the medium was removed, and the formazan was resuspended in DMSO supplemented with 0.57% acetic acid and 10% SDS (Sigma-Aldrich, St. Louis, MO, USA). Absorbance was measured at 570 nm by an ELISA reader (Elx 800, BioTek, San Diego, CA, USA) [32].

### 4.9. Anti-Promastigote Assay

The selected plant extracts were screened for antileishmanial activity by the MTT assay [66]. Promastigotes of *Leishmania tropica* were cultured in Medium-199 supplemented with 10% fetal bovine serum (FBS, Sigma-Aldrich, St. Louis, MO, USA), 100 IU/mL penicillin G, and 100 μg/mL streptomycin sulfate (Sigma-Aldrich, St. Louis, MO, USA) and incubated at 24 °C for 6–7 days until the culture count reached from 4–5 million/mL. The culture was counted using the Neubauer chamber during the log phase. Plant extracts were taken in a 96-well plate in three different concentrations: 1000, 500, and 250 µg/mL in triplicate. Amphotericin B (Sigma-Aldrich, St. Louis, MO, USA) was used as a reference drug, while DMSO (0.5%), being non-toxic to leishmanial parasite growth, was used as a negative control. A 100 µL of the complete culture (10^6^ cells/mL) was seeded to the 96-well plate already containing the screening samples at three different concentrations. The plate was incubated for 72 h at 25 °C. The viability of the promastigotes of all compounds was assessed by the MTT colorimetric method; 100 µL of the MTT dye was added to each well and re-incubated for 3 h at 37 °C. Finally, 40 µL of the DMSO was added as a stop solution. The absorbance was recorded using an ELISA plate reader (Elx 800, BioTek, San Diego, CA, USA) at 570 nm. The percent cytotoxicity of the extracts was calculated using the given formula:

(3) $$\mathrm{Percent}\;\mathrm{cytoxicity}=\frac{A570\;\mathrm{nm}\;\mathrm{of}\;\mathrm{the}\;\mathrm{Treated}\;\mathrm{sample}}{A570\;\mathrm{nm}\;\mathrm{of}\;\mathrm{Control}\;\mathrm{sample}}\times 100$$

IC_50_ (half-maximal inhibitory concentration) values of the test samples with ≥50% cytotoxicity were calculated by using percent hemolysis.

### 4.10. Statistical Analysis

All the analyses were performed in triplicate, and values were represented as mean (*n* = 3) ± SD. For qPCR analysis, three biological and three technical replicates were used for each sample. One-way analysis of variance (ANOVA) was used to find the significance of the difference between wild-type control plants and transgenic lines through GraphPad Prism Software Version 7.0 (GraphPad Prism^®^ Software, Inc. San Diego, CA, USA). *p* ≤ 0.05 was considered statistically significant. PAST 4.03 statistical software was used for principal component analysis (PCA) of FTIR spectra. LD_50_ of the test samples was calculated using table curve 2D v5.01 software.

## 5. Conclusions

In conclusion, present findings suggest that transgenic *A*. *bracteosa* plants are a rich source of metabolites that correlate with *rolB* gene expression. Generally, all transgenic lines showed significant antihemolytic, brine shrimp cytotoxic, anticancer, and antileishmanial activity. Overall, these results suggest that *A. bracteosa* extracts can be employed as futuristic possibilities for medicinal plants as a replacement to current anti-leishmanial and anticancer therapies. However, further studies are needed to uncover the potential molecular mechanism of *A*. *bracteosa* against these aggressive diseases.

## Figures and Tables

**Table 1:** FTIR analysis and functional groups.

No.	Functional Groups of Active Components in Crude Extracts							
	WT		ABRL1		ABRL2		ABRL3	
	Functional Groups	Peak Value	Functional Groups	Peak Value	Functional Groups	Peak Value	Functional Groups	Peak Value
1	Primary/Secondary amines	3287.70	Primary/Secondary amines	3270.74	Primary/Secondary amines	3272.71	Primary/Secondary amines	3283.41
2	Alkane	2918.21	Alkane	2917.51	Alkane	2917.72	Alkane	2918.39
3	Carboxylic acid, alkane	2850.07	Carboxylic acid, alkane	2849.59	Carboxylic acid, alkane	2849.76	Carboxylic acid, alkane	2850.21
4	Amino acid	1631.70	Ketone, carboxylic acid	1710.23	Ketone, carboxylic acid	1708.47	Thiol/mercaptan	2349.30
5	Alkane, Nitro compounds	1376.57	Amino acid	1594.74	Amino acid	1623.07	Amino acid	1625.01
6	Fluoride, alkyl halide	1248.95	Aromatic	1414.52	Alkane, Nitro compounds	1375.99	Alkane	1406.24
7	Alcohol, aliphatic amines	1029.22	Alkane, Nitro compounds	1373.30	Fluoride, alkyl halide, aliphatic amines	1241.37	Fluoride, alkyl halide	1249.00
8	p-substituted alcohols/phenols	815.80	Fluoride, alkyl halide, aliphatic amines	1237.64	Ether	1148.72	Ether	1147.89
9	Aromatic compound, primary/secondary amines	776.54	Alcohol, aliphatic amines	1014.17	Alcohol, aliphatic amines	1026.78	Alcohol, aliphatic amines	1026.83
10	Alkynes, alkyl halide, Aryl disulfide	518.90	p-substituted alcohols/phenols	812.42	p-substituted alcohols/phenols	815.76	Primary, secondary amines	866.90
11			Alkynes, alkyl halide, Aryl disulfide	525.19	Aromatic compound, primary/secondary amines	776.27	p-substituted alcohols/phenols	815.63
12					Alkynes, alkyl halide, Aryl disulfide	520.25	Aromatic compound, primary/secondary amines	776.14
13							Alkynes, alkyl halide, Aryl disulfide	517.93

**Table 2:** Antibacterial activity of *A*. *bracteosa*.

Samples	Zone of Inhibition at 100 µg/Disc (mm)							
	*M. luteus*	MIC (µg/mL)	*S. aureus*	MIC (µg/mL)	*E. aerogenes*	MIC (µg/mL)	*E. coli*	MIC (µg/mL)
WT	7.3 ± 0.2 ^e^	-	6.9 ± 0.3 ^f^	-	6.5 ± 0.1 ^f^	-	7.1 ± 0.4 ^f^	-
ABRL1	13.7 ± 0.6 ^c^	>100	15.1 ± 0.5 ^b,c^	>100	8.3 ± 0.2 ^d^	-	13.9 ± 0.4 ^c^	>100
ABRL2	13.8 ± 0.5 ^c^	>100	15.5 ± 0.4 ^b^	>100	8.9 ± 0.4 ^d^	-	14.5 ± 0.3 ^c^	>100
ABRL3	16.1 ± 0.6 ^b^	>100	16.9 ± 0.7 ^b^	>100	8.2 ± 0.3 ^d^	-	16.2 ± 0.5 ^b^	>100
kanamycin	26.2 ± 1 ^a^	0.31	25.1 ± 2 ^a^	0.33	23.7 ± 1 ^a^	0.29	24.4 ± 3 ^a^	0.30

WT = in vitro grown untransformed *Ajuga bracteosa* plant extract; ABRL1–3 = crude extracts of transgenic lines 1, 2, and 3 of *A. bracteosa*; kanamycin = positive drug; MIC = minimum inhibitory concentration. Data are represented as mean ± SD (*n* = 3). The values with different superscript (a–f) letters show significantly (*p* < 0.05) different means.

**Table 3:** Hemolytic activity of *Ajuga bracteosa*.

Samples	% Hemolysis			CC_50_ (µg/mL)
	Test Concentrations (µg/mL)			
	1000	500	250	
WT	16.25 ± 2 ^b^	5.25 ± 0.9 ^c^	2.97 ± 1 ^d^	1952.63 ± 12
ABRL1	9.76 ± 1 ^b,c^	4.01 ± 5 ^c,d^	0.82 ± 0.07 ^e^	5309.04 ± 13
ABRL2	8.43 ± 0.8 ^b,c^	2.87 ± 6 ^d^	2.72 ± 0.3 ^d^	6179.54 ± 9
ABRL3	6.28 ± 0.3 ^c^	2.41 ± 4 ^d^	0.88 ± 0.1 ^e^	7293.05 ± 7
AAPH	100 ^a^			0.07

WT = in vitro grown untransformed *Ajuga bracteosa* plant extract; ABRL1–3 = crude extracts of transgenic lines 1, 2, and 3 of *A. bracteosa*; AAPH = positive control; CC_50_ = 50% cytotoxic concentration. Data are represented as mean ± SD (*n* = 3). The values with different superscript (a–e) letters show significantly (*p* < 0.05) different means.

**Table 4:** Brine shrimp cytotoxicity of *Ajuga bracteosa*.

Samples	% Mortality After 24 h			LD_50_ (µg/mL)
	Test Concentrations (µg/mL)			
	200	66	21.8	
WT	70.33 ± 3 ^b^	50.4 ± 2 ^d^	30.3 ± 3 ^f^	75.6 ± 9
ABRL1	79.3 ± 5 ^a^	60.3 ± 2 ^c^	40.0 ± 3 ^e^	39.6 ± 4
ABRL2	68.6 ± 4 ^b^	58.6 ± 1 ^c^	40.6 ± 2 ^e^	41.62 ± 2
ABRL3	67.14 ± 5 ^b^	59.2 ± 3 ^c^	42.3 ± 4 ^e^	43.62 ± 5
Doxorubicin	84.65 ± 10 ^a^			5.8 ± 0.3

WT = in vitro grown untransformed *Ajuga bracteosa* plant extract; ABRL1–3 = crude extracts of transgenic lines 1, 2, and 3 of *A. bracteosa*; Doxorubicin = positive control; LD_50_ = median lethal dose. Data are represented as mean ± SD (*n* = 3). The values with different superscript (a–f) letters show significantly (*p* < 0.05) different means.

**Table 5:** IC_50_ values of cell proliferation inhibition of *Ajuga bracteosa* extracts.

Treatment	IC_50_ (µg/mL)					
	HepG2	LM3	A549	HT-29	MCF-7	MDA-MB-231
WT	>100	>100	>100	>100	>100	>100
ABRL1	71.2 ± 3.1 ^d^	58.3 ± 2.3 ^c^	98.1 ± 2.4 ^f^	98.2 ± 3.3 ^f^	>100	>100
ABRL2	79.5 ± 4.6 ^d,e^	53.4 ± 1.5 ^c^	94.2 ± 1.7 ^f^	99.7 ± 1.9 ^f^	>100	>100
ABRL3	57.1 ± 2.2 ^c^	46.2 ± 1.1 ^b^	72.4 ± 1.3 ^d^	73.3 ± 2.1 ^d^	98.7 ± 1.6 ^f^	97.1 ± 2.5 ^f^
Ellipticine	0.38 ± 0.01 ^a^	0.47 ± 0.04 ^a^	0.41 ± 0.05 ^a^	0.39 ± 0.04 ^a^	0.35 ± 0.02 ^a^	0.45 ± 0.03 ^a^

WT = in vitro grown untransformed *Ajuga bracteosa* plant extract; ABRL1–3 = crude extracts of transgenic lines 1, 2, and 3 of *A. bracteosa*; Ellipticine = standard anticancer drug; IC_50_ = half-maximal inhibitory concentration. Data are represented as mean ± SD (*n* = 3). The values with different superscript (a–f) letters show significantly (*p* < 0.05) different means.

**Table 6:** Antileishmanial activity of *Ajuga bracteosa*.

Samples	% Mortality after 72 h			IC_50_ (µg/mL)
	Test Concentrations (µg/mL)			
	1000	500	250	
W	63.52 ± 7 ^d^	55.64 ± 4 ^e^	47.23 ± 2 ^f^	313.99 ± 6
ABRL1	78.14 ± 1 ^b^	67.38 ± 1 ^c^	56.63 ± 5 ^e^	163.04 ± 8
ABRL2	75.48 ± 6 ^b^	68.42 ± 6 ^c,^	61.69 ± 6 ^d^	77.53 ± 7
ABRL3	74.53 ± 8 ^b^	69.48 ± 4 ^c,^	62.14 ± 3 ^d^	56.16 ± 2
Amphotericin B	100 ^a^			0.01

WT = in vitro grown untransformed *Ajuga bracteosa* plant extract; ABRL1–3 = crude extracts of transgenic lines 1, 2, and 3 of *A. bracteosa*; Amphotericin B = positive drug; IC_50_ = half-maximal inhibitory concentration. Data are represented as mean ± SD (*n* = 3). The values with different superscript (a–f) letters show significantly (*p* < 0.05) different means.

**Table 7:** The gene primer sequences.

Genes	GenBank Accession No.	Primer Sequences	Size (bp)
House Keeping Genes			
*βActin*	DQ531565	F: GATTGAGCACGGTATTGTTAG R: ACACCATCACCAGAATCCAAC	259
*18s*	HQ730915.1	F: GGAGAGGGAGCCTGAGAAAC R: GATTTAGATTGTACTCATTCC	122
*Agrobacterium rhizogenes* genes			
*rolB* (qPCR)	X03433	F: CGAGGGACTGAAAACCGCC R: CCGAGAGTCGCAGGGTTAG	127
*rolB* (Simple PCR)	X15952.1	F: GCTCTTGCAGTGCTAGATTT R: GAAGGTGCAAGCTACCTCTC	779

## Data Availability

The datasets used and/or analyzed during the current study are available from the corresponding author on reasonable request.

## Sample Availability

Samples of plant material are available from the authors.
